# Application of Ultra-Small Micro Grinding and Micro Milling Tools: Possibilities and Limitations

**DOI:** 10.3390/mi8090261

**Published:** 2017-08-24

**Authors:** Benjamin Kirsch, Martin Bohley, Peter A. Arrabiyeh, Jan C. Aurich

**Affiliations:** Institute for Manufacturing Technology and Production Systems, University of Kaiserslautern, P.O. Box 3049, 67653 Kaiserslautern, Germany; martin.bohley@mv.uni-kl.de (M.B.); Peter.Arrabiyeh@mv.uni-kl.de (P.A.A.); fbk@mv.uni-kl.de (J.C.A.)

**Keywords:** micro pencil grinding tools, micro end mills, electroless plating

## Abstract

Current demands for flexible, individual microstructures in high quality result in high requirements for micro tools. As the tool size defines the minimum structure size, ultra-small tools are needed. To achieve tool diameters of 50 µm and lower, we investigate the complete manufacturing chain of micro machining. From the development of the machine tools and components needed to produce and apply the micro tools, the micro tools themselves, as well as the micro machining processes. Machine tools are developed with the possibility of producing the micro geometry (cutting edge design) of micro tools as well as plating processes to produce super abrasive micro grinding tools. Applying these setups, we are able to produce ultra-small micro grinding and micro milling tools with typical diameters of 50 µm and down to 4 µm. However, the application of such tools is very challenging. The article presents possibilities and limitations in manufacturing the micro tools themselves as well as microstructures made with these tools. A special emphasis will be on the influence of the tool substrate in micro milling and grain sizes in micro grinding.

## 1. Introduction

A component’s surface characteristics are crucial for its functionality, durability, and the quality at which it performs the task it is designed for. The surface of a component determines how the component interacts with its environment [1]. This interaction can be demonstrated by changing the corrosive nature of a steel workpiece by adding alloying elements or coating its surface [2]. Heat treatment can increase a components hardness, change its metallurgical microstructure or reduce residual stresses [3]. Another way to influence the mechanical and physical properties of a component is manufacturing geometrically defined microstructures on its surface [4]. The wear of components such as chain joints can be drastically reduced by machining microstructures into their chain pins. The microstructures on the pins can hold lubricant, increase its wettability, and in turn reduce the joint friction between the two components [5]. Micro structures can also influence the mechanical properties of micro components. A study conducted by Godart et al., showed that 50 µm wide micro milled microstructures with a depth of 10–20 µm could increase tensile strength and decrease the fracture elongation in commercially pure-titanium workpieces [6].

Micro components with functionally optimized surfaces are used in bioreactors to increase bacterial adhesion and increase bacterial growth [7] or in medical applications to increase the lifetime of implants or cardiac pacemakers [8]. The precision industry takes advantage of these properties to manufacture lighter and smaller micro parts with more functions for given product size [9]. To meet the demand of precision industries such as the biomedical, telecommunication, aerospace, and electronics industries, processes are modified or new processes developed [10]. With regard to economic success, both technological advantages as well as a competitive price level are necessary. Processes like etching, micro molding and Focused Ion Beam (FIB) are used to mass produce micro parts and components [11]. Due to the high accuracy, low achievable surface roughness, and very high geometrical flexibility of produced microstructures, machining processes like micro milling [12] and micro grinding are much more suitable for small batch production on the micron scale compared to mass production processes [13]. 

Micro pencil grinding tools (MPGTs) and single edged micro end mills made of cemented carbide and manufactured via precision grinding are available in various geometrical shapes and can be scaled down to 4 µm for micro pencil grinding tools [14] and 10 µm for micro end mills [15]. Micro pencil grinding tools consist of a cemented carbide basic body and an abrasive body. The abrasive body consists of superabrasive grits (diamond or cubic boron nitride (cBN)) and the bond; in this case the bond is a nickel coating. Micro grinding uses much lower feed rates than micro milling, but covers harder and brittle materials [11]. 

No references exist that evaluate the influence of the cemented carbide grain size on the performance of micro end mills. This paper will deal with the influence of the tungsten carbide grain size on the applicability of micro end mills with effective milling cutter diameters of 50 µm. Concerning micro grinding, the electroless plating method to produce the abrasive body will be evaluated. While the plating method itself is not new, only little reference can be found where it was applied to micro grinding tools [16]. The influence of the size of the abrasive grits on the performance of MPGTs and the boundaries of electroless plating will be evaluated. 

## 2. Materials and Methods

### 2.1. Micro Milling Center

The manufacturing and the application of the ultra-small micro end mills (USM-mills) [17] was conducted on a precision three axes machine tool. This machine tool, called micro milling center (MMC) [18], was developed at our lab. The key aspect of this machine tool is that it enables to produce and use a micro end mill without reclamping. Thus, the run-out error is reduced by the reclamping error. The design of the machine tool follows the idea of a small machine tool for the production of small parts and structures. The whole machine tool is desktop sized covering an installation space of only 760 mm × 675 mm × 500 mm. Due to this small size, small axes with low travel and low moving masses could be implemented, resulting in high velocities and low energy consumption.

**Figure 1:**
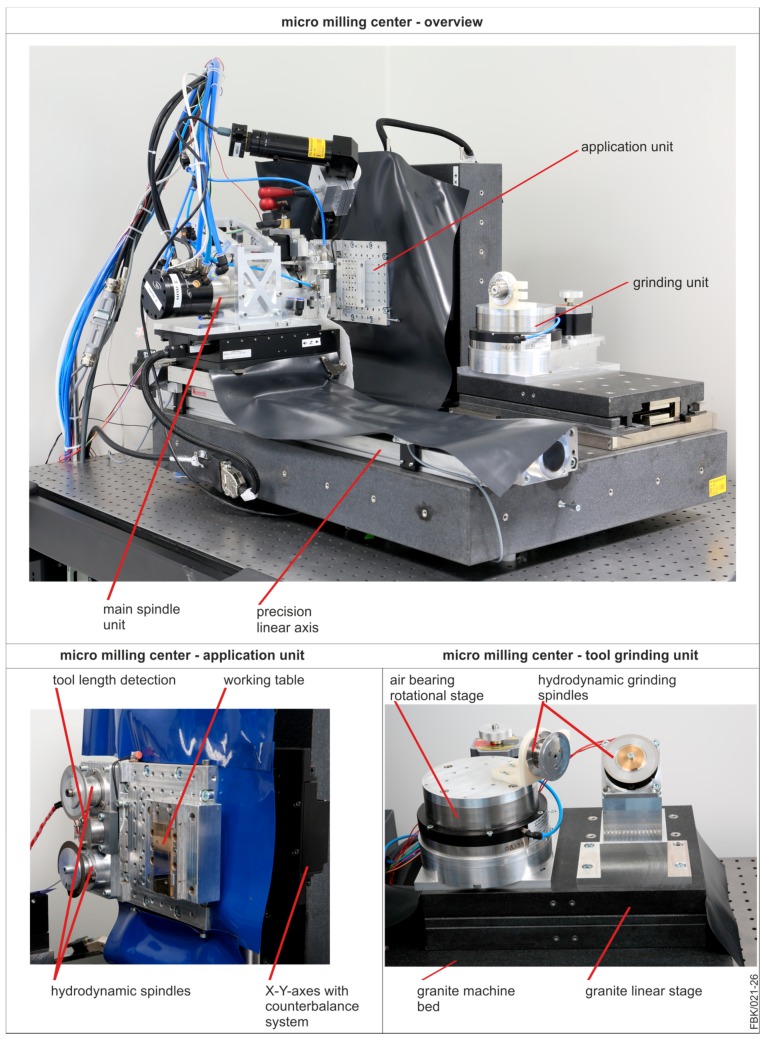
Micro milling center (MMC) after [18].

The machine tool consists of three functional units: the main spindle unit, the tool grinding unit, and the application unit (Figure 1). The main spindle unit is mounted on a precision linear axis with a travel of 560 mm connecting the tool grinding unit and the application unit. The main spindle itself is mounted on an Aerotech ANT130-110-L-Plus (Aerotech Inc, Pittsburgh, PA, USA) axis with a travel of 110 mm, a resolution of 1 nm and a positioning accuracy of ± 0.25 µm. This axis conducts the precise Z-movement (in axial spindle/tool direction). The main spindle is an ABL MM125 (Air Bearings Ltd, Poole, UK)air bearing spindle with a maximum rotational speed of 125,000 rpm and a run-out of 2–4 µm in a range of 10,000 to 45,000 rpm. The spindle as well as the spindle mount is liquid cooled with a temperature stability of 0.1 K. For manufacturing the tools (compare Section 2.3), the spindle can be precisely positioned (rotatory) with a stepping motor and a belt drive.

The tool grinding unit is built on a granite linear stage with a travel of 150 mm, a resolution of 1 nm and an accuracy of ±0.3 µm. On this granite stage, an air bearing rotation stage is mounted which can carry up to four hydrodynamic spindle motors with diamond grinding wheels for tool manufacturing. These spindle motors are powered by 6.4 W brushless DC motors (BLDC) and have a maximum rotational speed of 12,500 rpm and a run-out error of <0.8 µm peak-to-peak.

The application unit is mounted on two Aerotech ANT130-110-L Plus axes. As the Y-axis moves in a vertical direction, it needs to be fitted with Airpel (Airpot Corporation, Norwalk, CT, USA) air bearing counterweights to limit the needed motor power and to reduce heat generation for a higher machining precision. Both axes have a travel of 110 mm, a resolution of 1 nm, and a positioning accuracy of ±0.375 µm.

### 2.2. Precision 4-Axes Machine

**Figure 2:**
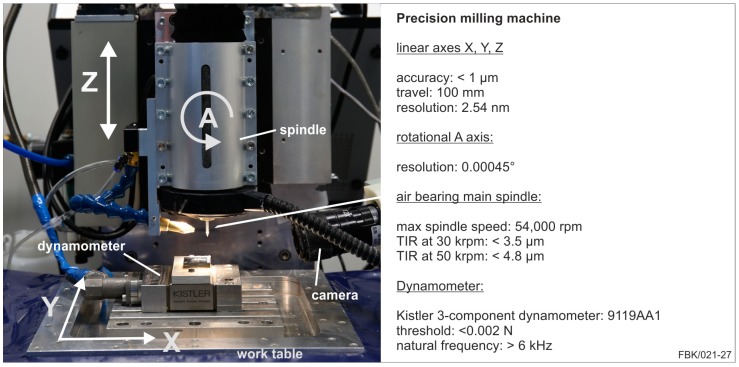
Precision 4-axes machine after [19].

The precision 4-axes machine tool was used for the application of the micro pencil grinding tools (MPGTs), Figure 2. This machine tool is built up on a massive granite bed with a moving table design. This table consists of two air bearing axes in X- and Y-direction. They are powered by stepper motors and ball screws at a resolution of 2.54 nm. With this configuration, a travel of 100 mm in each direction with a positioning accuracy of <1 µm is possible. The Z-axis carries the rotational axis as well as the air bearing main spindle. This Z-axis is cross roller bearing guided and driven with a stepper motor in combination with a ball screw. The travel is 100 mm with a positioning accuracy of <1 µm. The rotational axis is a harmonic drive servo system with a resolution of 0.00045°. With this axis, the main spindle can be tilted in a range of ±30°. The air bearing main spindle has a maximum spindle speed of 54,000 rpm. The run-out is rising from 3 µm at the minimal spindle speed of 5000 rpm to 6 µm at 54,000 rpm [19].

### 2.3. Manufacturing of Micro End Mils (MEMs)

**Figure 3:**
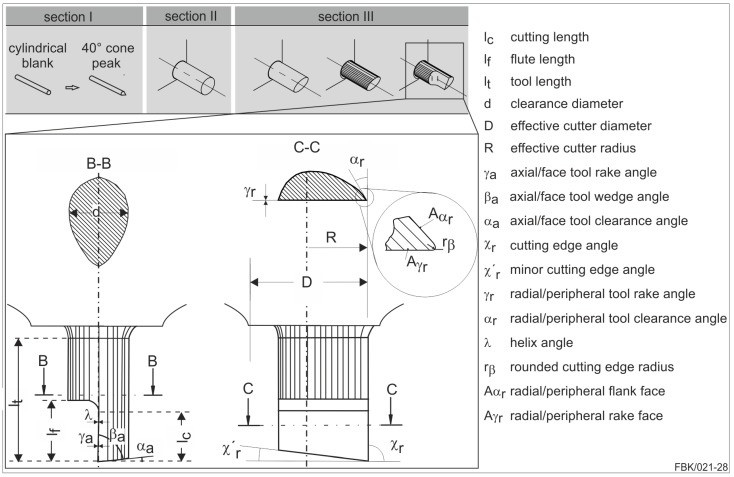
Schematic of the Tool grinding process and nomenclature after [15,17].

The micro tools were manufactured via grinding. Advantages of grinding are the short process time, the excellent achievable quality, and small diameters of the micro tools. The manufacturing was done according to the method proposed in [15], compare Figure 3. In Section I, a cylindrical tungsten carbide tool blank is ground with a 40° cone at the top on a conventional tool grinding machine tool. All subsequent steps are conducted on the MMC described in Section 2.1. The pre-grinding of the tip cylinder (Section II) is conducted with a diamond grinding wheel (mesh #800, width 250 µm, diameter 58 mm). The tip cylinder of the pre-grinding process is adapted to the desired tool diameter in this case 50 µm. With a fine-grained diamond grinding wheel (mesh #4800, width 50 µm, diameter 58 mm), the final single edge micro end mill is fabricated (Section III). In this step, the actual cutting tool geometry, namely the cutting edge geometry, the flank, and the rake face, etc., are manufactured. The faceting of the flank face is adjusted to the intended feed per tooth and the run-out of the spindle to assure that the flank face does not contact the workpiece. 

### 2.4. Manufacturing of Micro Pencil Grinding Tools (MPGTs)

MPGTs, just like common high performance grinding wheels, consist of a basic body and the abrasive body; the abrasive body consist of the abrasive grains or grits and the metallic bond [20]. A number of methods have been used to bind these abrasive grits to the basic body. One of the more commonly used ones is a sintering method that uses high temperatures and pressures up to 20 MPA to manufacture abrasive layers that bind the abrasive grits with a metallic matrix (commonly bronze). The metallic matrix covers the abrasive grits completely. Thus, a dressing process is required to create the required grit protrusion [21]. The process can manufacture micro pencil grinding tools down to 180 µm [22].

Gäbler et al. used an chemical vapor deposition method (CVD) to manufacture an abrasive layer for micro grinding tools by depositioning a synthetic, polycrystalline diamond layer from a gas atmosphere [23]. This method can produce tools with much smaller diameters, Hoffmeister et al. manufactured micro pencil grinding tools with diameters down to 50 µm. In addition, the method can be applied to diverse basic body geometries. The diamond crystals are fine grained and have sharp cutting edges that cannot be produces using other methods [24]. However, this leads to rapid tool clogging and in turn to tool breakage [25].

Another option for manufacturing micro pencil grinding tools can be achieved by binding the abrasive grits to the abrasive with an electrolytic nickel layer. Two methods have been used to manufacture these tools; an electroplating method [26] and an electroless plating method [16]. In both methods the tool is immersed into a plating solution containing nickel ions (Ni+^2^). The surface of the basic body needs to provide with the missing electrons in order to manufacture a nickel layer [27]. Electroplating connects the basic body to an electric cycle in which the tool functions as a cathode and a nickel resource that is immersed in the plating solution functions as an anode. Once a current is provided, an electrical field is produced, causing the nickel ions to gravitate towards the basic body. In addition, the anode provides electrons to the basic body while replenishing the plating solution with new nickel ions [28]. Electroless plating provides uses an reducing agent to provide nickel affine surfaces with the missing electrons [16]. The adjustable grit protrusion for both methods eliminates trueing and dressing steps that are necessary for other plating methods like sintering. However, for this paper the electroless plating method was chosen because it generally produces more uniform abrasive layers with a higher binder hardness [27].

**Figure 4:**
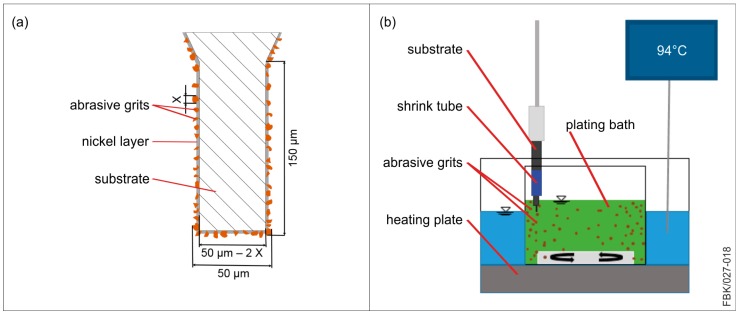
(**a**) Schematic of electroless plated MPGT tip; (**b**) schematic of electroless plating setup.

For this paper, the basic body introduced in Figure 3 (cylindrical tungsten carbide tool blank) is used. The MPGTs undergo the same procedure as the MEMs up to Section II (compare Section 2.3) to a final length of the tip cylinder of 150 µm. The diameter of the tip cylinder is adjusted to the process and the grit size of the MPGT. For instance, when a final diameter of 50 µm is to be manufactured with an average grit size of 2 µm and a monolayer is to be achieved, a diameter of 46 µm is ground on the tip cylinder (see Figure 4a). 

The abrasive body is manufactured via electroless plating. This is done in the plating bath, containing all ingredients of the abrasive body to be manufactured. Before electroless plating, the substrate is degreased at a temperature of 80 °C in a 200 g/L sodium hydroxide solution, etched in a 1.5 g/L hydrochloric acid solution for cleaning, and then electroplated with a thin adhesive nickel layer in a solution made of 250 g/L nickel chloride and 10 g/L hydrochloric acid. The thin nickel layer is necessary for the tungsten carbide surface to respond quicker to the following electroless plating. Sodium hypophosphite is used to reduce the metal ions in the plating solution to enable the metal deposition onto the substrate. A detailed description of the chemical background can be found in [27]. Table 1 lists the components of the plating solution and Figure 4b shows the experimental setup for plating. The grits are whirled up using a magnetic stirrer with a rotation speed of 60 rpm. The grits adhere to the substrate surface and are coated with a nickel layer. The substrate rotates at 1–3 rpm (lower values for larger grit sizes) to gather grits on the whole circumference [27].

There are two characteristic values of the abrasive body: the grit concentration (grits per area) and the grit protrusion (to assure chip space and space for the metal working fluid). The grit concentration is controlled by the amount/concentration of grits in the plating bath, and the grit protrusion by the embedding time. Using the components listed in Table 1, a nickel growth of 21 µm/h was measured. The embedding time can hence be calculated to reach the desired plating thickness and grit protrusion. Common values taken from macro grinding wheels are plating thicknesses of 50–70% of the average grit size. This value is a balance between grain retention forces and sufficient chip space. Plating times and grit concentrations are listed in Section 3.2. The plating time consists of the plating time needed to gather the grits for the tool (main plating time) and the time needed to adjust the grit protrusion (embedding time). During the embedding step, the stirring motion in the plating solution is turned off to stop the grit motion in the beaker, so no further grains adhere to the substrate. 

## 3. Results

### 3.1. Electroless Plating of Micro Pencil Grinding Tools

**Figure 5:**
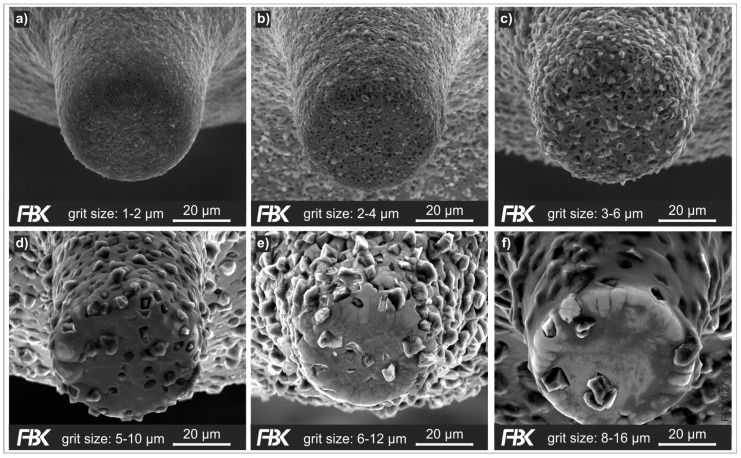
Electroless plated MPGTs with grit sizes (**a**) 1–2 µm; (**b**) 2–4 µm; (**c**) 3–6 µm; (**d**) 5–10 µm; (**e**) 6–12 µm, and (**f**) 8–16 µm.

MPGTs with different grit sizes were produced. The substrates (tool blanks) were made of tungsten carbide (WC content: 92%, Co content: 8%, grain size: 0.2 µm). Figure 5 shows MPGTs with different grit sizes; Table 2 lists the plating parameters used to manufacture these tools. For each nominal grit size, the diameter of the substrates was prepared to reach an effective diameter of ~50 µm after plating.

The electroless plating process is limited by the solution lifetime. We observed that a solution can spontaneously decompose after 60–120 minutes, completely depleting the nickel ions in the solution. This time frame can be influenced by the thiourea concentration in the solution. It was documented that a larger thiourea concentration increases the lifetime of the solution, but negatively influences the plating quality and the nickel growth. A too high thiourea concentration can inhibit the nickel growth completely [27]. This time limitation complicates the plating process for bigger grit sizes, as those necessitate higher embedding times.

Generally speaking, the grit size is the greatest limitation to the plating process. Plating tools with smaller grits is good to control; the grits have a larger area to populate at the substrate. This can be seen in Figure 5. The uniformity of the grit distribution diminishes with rising grit sizes. The biggest examined grit size, 8–16 µm, is too large for the given substrate diameter of 36 µm to achieve a feasible grit concentration. In addition, larger grits require a much larger grit concentration increasing the costs of the manufacturing process tremendously. The minimal grit concentration required to manufacture an MPGT with a grit size of 8–16 µm is 23 g/L. In comparison, we found that for a grit size of 1–2 µm, a concentration of 0.15 g/L is sufficient to manufacture an MPGT with a diameter of 50 µm [27]. Experimental results revealed the grit size of 6–12 µm (Figure 5e) to be the largest grit size to deliver MPGTs with a sufficient grit concentration.

### 3.2. A Case Study in Micro Grinding

**Figure 6:**
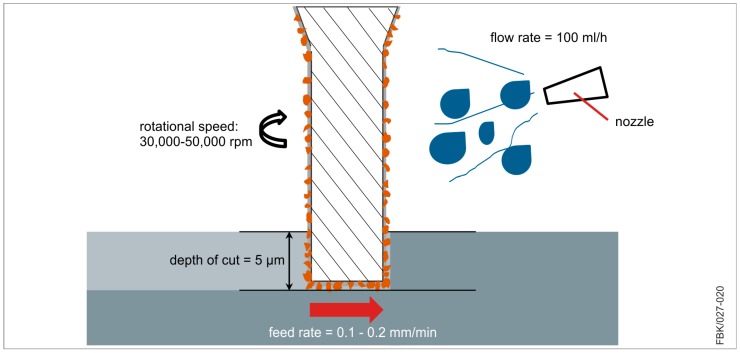
Micro grinding process kinematics.

The grit size not only is the most crucial factor for the manufacturing of the MPGTs, but also for their application. To demonstrate this, MPGTs coated with the grit sizes 1–2 µm and 3–6 µm were applied with different process parameters in a case study (see Table 3). The workpiece material was 16 MnCr5, hardened to 665 ± 15 HV30. After clamping, the workpiece was face ground to achieve maximum evenness. A water-soluble metal working fluid was applied at a flow rate of 100 mL/h. Figure 6 shows the process kinematics for the case study.

**Figure 7:**
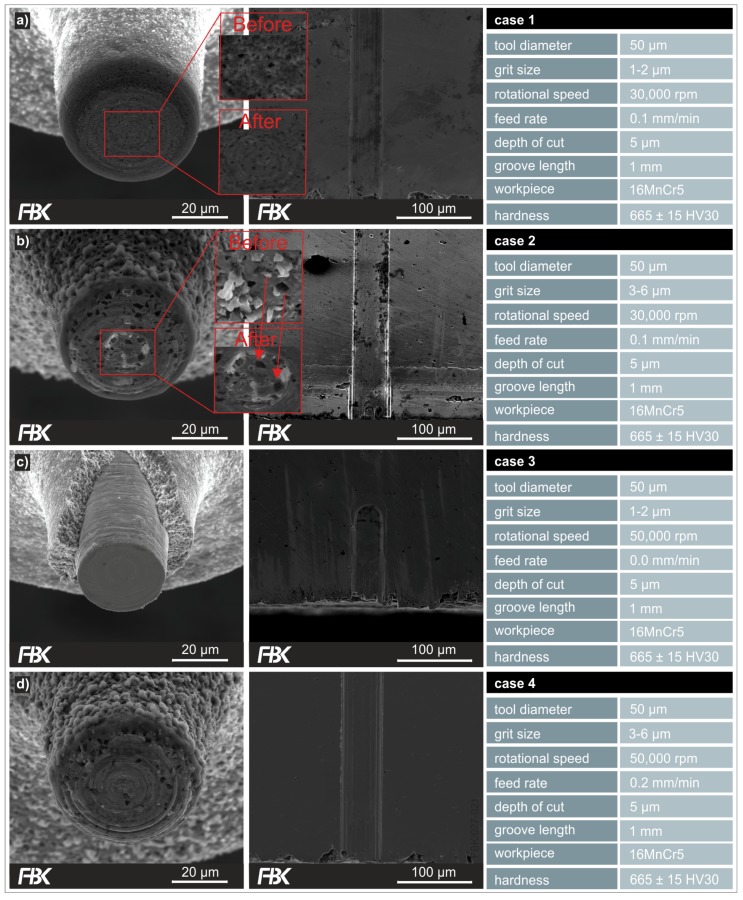
MPGTs after machining (**left column**), entry area of grooves (**middle column**) and process conditions (**right column**), for case 1 (**a**), case 2 (**b**), case 3 (**c**) and case 4 (**d**).

The experiments were performed on the precision milling machine presented in Figure 2. Grooves with a length of 1 mm at a depth of cut of 5 µm were machined for each case presented. All experiments and measurements were done thrice. Figure 7 shows the MPGTs after machining and the entry point in the workpiece of the respective grooves.

**Figure 8:**
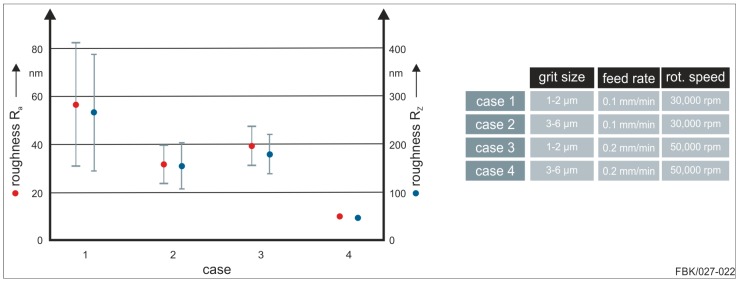
Bottom surface roughness of first 100 µm groove length.

The cases 1, 2, and 4 were performed successfully with all nine tools (including statistical repetitions) intact after machining the grooves (Figure 7a,b,d). In contrast, only one out of the three tools of case 3 managed to machine the complete groove length successfully. Figure 7c shows one of the MPGTs that failed to complete the entire groove length. That is, the MPGTs with grit sizes of 1–2 µm perform poorly at higher feed rates and higher rotational speeds. Higher feed rates result into higher chip thicknesses and hence higher loads of the grains and the abrasive body. In that sense, the negative influence of higher feed rates on the performance of the tools is expectable. In contrast, higher rotational speeds result into higher cutting speeds and hence lower chip thicknesses, commonly improving machining conditions in grinding. However, with the given kinematics the cutting speed drops to zero towards the center of the tools. The grits at the center of the tool tip hence only rub and plough and generate heat. At higher feed rates, the grits towards the center of the tool tip become more loaded, obviously resulting in bond overload. Another factor is the thermal expansion coefficient. This coefficient is much larger for the nickel bond than for cemented carbide basic body, causing stresses between the substrate and the bond. Obviously, all that is compensated by larger grit sizes, as case 4 performed well, which can also be seen in the resulting bottom surface roughness of the grooves (Figure 8).

The bottom surface roughness of the grooves was measured at the respective entrance are for the four cases (Figure 8). The measurement was done according to DIN EN ISO 4287 [29], i.e., a measurement length of 400 µm in direction of the maximum roughness profile was maintained; except for case 3, where a lower measurement length had to be used as the groove length was shorter. Three main conclusions can be drawn: higher rotational speeds result in a more stable process (smaller standard deviations) and a lower surface roughness, and higher grit sizes result in a lower roughness.

The more stable behavior at higher rotational speeds may be caused by the spindle characteristics (characteristic excitation in the axial direction) rather than by tool specifications, and should not be overrated. A lower roughness at higher rotational speeds is the result of smaller uncut chip thicknesses, a well-known interdependency from macro grinding processes. A rather unusual result is the positive influence of higher grit sizes. In macro grinding, those result in a higher roughness due to a rising uncut chip thicknesses. Here, it is vice versa. This could be traced back to different material removal mechanisms. Possibly, the smaller grit sizes and chip space could result in a more ploughing-dominated material removal regime rather than cutting-dominated, thus diminishing the surface finish. Also, glazing of the tools could intensify the ploughing domain. Smaller grit sizes entail smaller chip spaces and hence are more prone to glazing. In addition, larger grits provide higher grain retention forces. Wear flats are generated on the grits resulting into smaller chip thicknesses and hence a lower surface roughness. The smaller grits break out before wear flats are generated and hence larger chip thicknesses result.

### 3.3. Manufacturing of Ultra Small Diameter Micro Milling Tools

**Figure 9:**
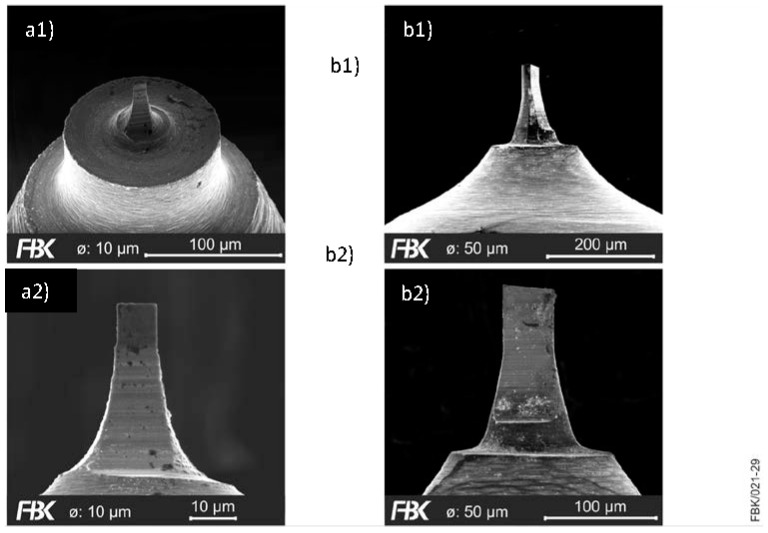
Micro End mills with different diameters (**a1** and **a2**) 10 µm (**b1** and **b2**) 50 µm at different magnifications.

To demonstrate the possibilities and limitations of manufacturing USM-mills, tools with an effective milling cutter diameter of 10 µm and 50 µm were manufactured (Figure 9). The detailed view reveals some limitations when reducing the tool diameter. While the tool with diameter 50 µm provides sharp cutting edges and the faces appear quite smooth, the cutting edges of the small diameter tool appear blunt and the grinding grooves can clearly be seen. This shows that the tool diameter cannot be reduced by only scaling the tool geometry. The tool’s micro geometry has to be adapted to the tool diameter. Also, the tool substrate (namely the grain size of the cemented carbide) and the manufacturing parameters have to be adjusted in order to master the occurring size effects.

### 3.4. A Case Study in Micro milling—Influnece of Cemented Carbide Specifications

In macro machining, the specification of the material a tool is made of has a high influence on the performance of the tool. In micro machining, this influence can be expected to be even higher, as the tool’s cutting edge has to be very sharp in micro machining. This is due to the fact that very small uncut chip thicknesses necessitate very small cutting edge rounding to avoid ploughing [30,31]. As there are very few investigations on the influence of the cemented carbide specifications on the performance of micro tools, a small case study will be presented to ascertain this.

**Figure 10:**
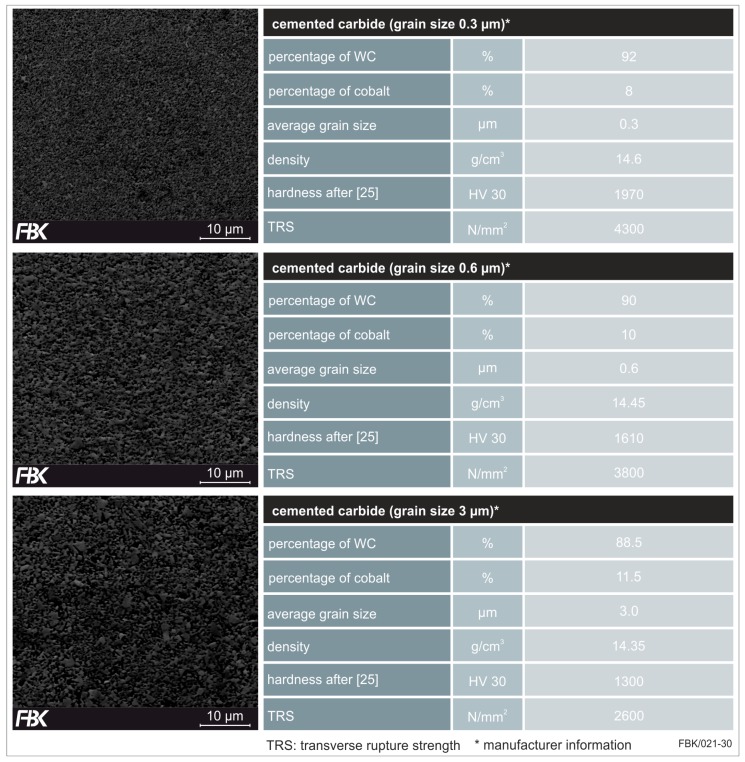
Specifications of cemented carbide for the different grain sizes of (**top**) 0.3 µm, (**middle**) 0.6 µm and (**bottom**) 3 µm

Three different specifications of cemented carbide were investigated, firstly differing in the grain size of the tungsten carbide content. Average grain sizes of 0.3 µm (ultrafine-grained), 0.6 µm (finest-grained), and 3 µm (coarse-grained) were considered. The specifications and properties, as well as a SEM (scanning electron microscopy)-image of the cross sections can be found in Figure 10. With rising grain size, the hardness as well as the transverse rupture strength (TRS) decline. In addition, the amount of the binder phase (cobalt) is rising. 

Milling tools were manufactured according to the approach described in Section 2.3 using the three types of cemented carbide. The single edge USM-mills with a flat face had an effective milling cutter diameter of 50 µm, a minor cutting edge angle of *χ*’_r_ = 12°, and were optimized for a spindle run-out of 3 µm as well as a feed per tooth *f*_z_ < 3 µm. 

**Figure 11:**
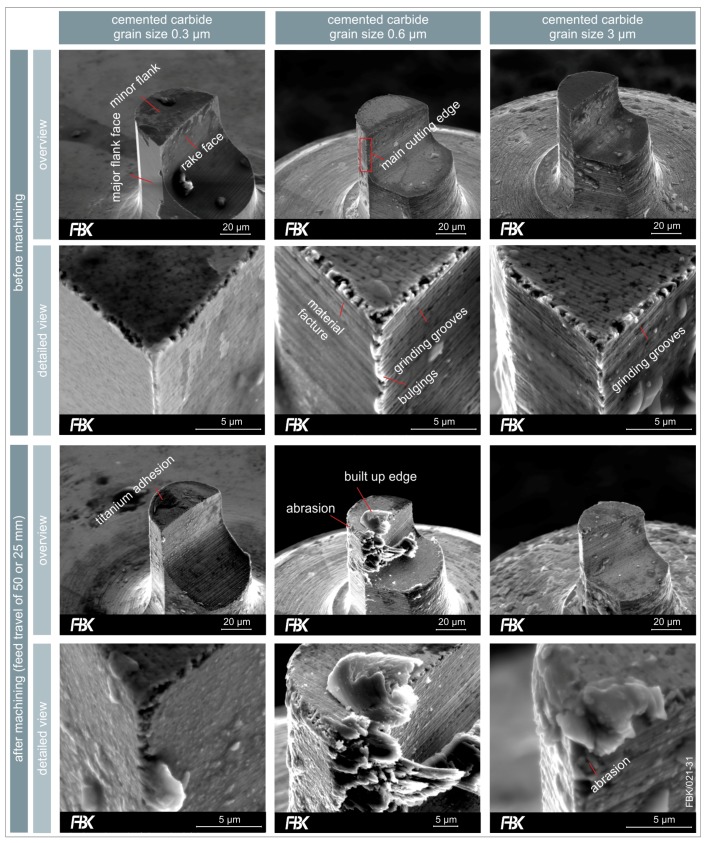
Influence of cemented carbide specifications on tool quality and wear in dependence of the grain size (from left to right: 0.3 µm, 0.6 µm and 3 µm) before (**top**) and after machining (**bottom**).

The influence of the grain size of the cemented carbide on the quality of the manufactured tools can be seen Fin Figure 11. The tools made of ultrafine-grained (0.3 µm) cemented carbide exhibit a very homogenous and smooth cutting edge geometry. The main cutting edge does not show any breakouts and the minor cutting edge only a few. The intended ideal geometry as depicted in Figure 3 was closely matched. In addition, virtually no grooves or pile-ups resulting froulm tool grinding resulted.

The finest-grained cemented carbide (0.6 µm) also results in a very homogenous shape and surface of the tool. However, in contrast to the ultrafine-grained cemented carbide, the tool exhibits grinding grooves resulting from its manufacturing. The main cutting edge shows some bulgings, being pile-ups from tool grinding. The minor cutting edge has some breakouts.

The coarse-grained cemented carbide exhibits a rough surface finish, grinding grooves can be seen very clearly. The main and the minor cutting edge show breakouts. 

**Figure 12:**
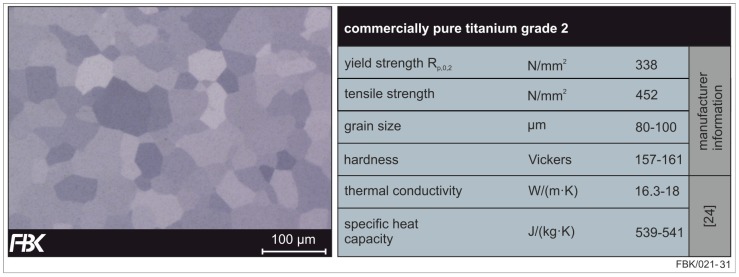
Etched cross-section (**left**) and properties of commercially pure (CP) titanium grade 2 (**right**) after [35].

The tools were employed in slot milling. The investigated material was commercially pure titanium grade 2 (maximum amount of iron Fe < 0.2% and oxygen O < 0.18%); specifications can be found in Figure 12. Titanium was chosen due to its chemical resistance [32] and good bio-compatibility [33], making it a widespread material in the aerospace and medical sectors. An example are pace makers [34], needing micro structures that could be manufactured by micro milling.

Grooves were milled at a cutting speed of *v*_c_ = 7.54 m/min at a feed per tooth of *f*_z_ = 1.2 µm and a depth of cut of 5 µm. The length of the grooves was 50 mm for the WC grain sizes 0.3 µm and 0.6 µm and 25 mm for the grain size of 3 µm. The tools were recorded via SEM after their application (Figure 11).

The wear of the ultrafine-grained (0.3 µm) cemented carbide tool was the lowest in this research. There is slight cutting edge rounding, but the tool is still capable of cutting. Some adhesions of titanium could be detected, but there was no build-up edge formation at the rake face. 

The finest-grained (0.6 µm) showed wear of the minor flank face. Especially the edges between the major and minor flank face were severely rounded. There was a considerable build-up edge formation on the rake face.

The coarse-grained (3 µm) cemented carbide tools were evaluated after a feed travel of 25 mm as no tool was able to reach 50 mm without failure. On the contrary, no build-up edge formation could be detected after 25 mm of feed travel and the wear of the flank and rake faces was very low. However, breakouts resulting from abrasion can be seen at the main cutting edge. It can be concluded that those cause the abrupt failure of those tools due to the rise of the cutting forces.

**Figure 13:**
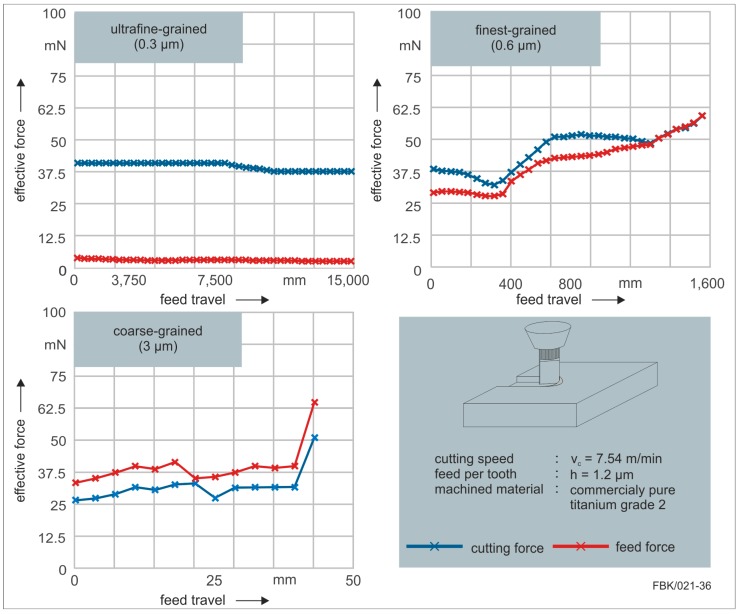
Forces in dependence of the cemented carbide specifications (top left: 0.3µm, top right: 0.6 mm and 3 mm (bottom left) when micro milling (process specifications bottom right).

To further evaluate the performance resulting from the cemented carbide specifications, forces were recorded during milling of the grooves with a dynamometer (Figure 13). The feed travel of the two finer grain sizes (0.3 µm and 0.6 µm) were extended; while that of the finest-grain size (0.6 µm) was extended to 1600 mm until tool failure, the feed travel of the ultrafine-grain size (0.3 µm) was extended up to 16,000 mm without failure. At that point, the experiment was interrupted. Some conclusions can easily be drawn by this investigation. First of all, the finer the grain size, the longer the achievable feed travel. Secondly, the finer the grain size the smaller the process forces. Especially, the feed force was considerably smaller for the ultrafine-grain size. This could be traced back to the tool manufacturing. As discussed, the intended ideal geometry of the tool was closely matched using the ultrafine-grained cemented carbide. As described in Section 2.3, the facettes on the flank face are adjusted to a certain feed per tooth. When this feed per tooth is exceeded, the flank face touches the workpiece, what can result in severe wear or abrupt tool failure. The data implies that this occurred for the finest-grained and coarse-grained cemented carbide tools. While the effective feed force was very low for the ultrafine-grained tools, those were considerably higher for the other two grain sizes. This can be traced back to the flank face contacting and pushing against the workpiece.

**Figure 14:**
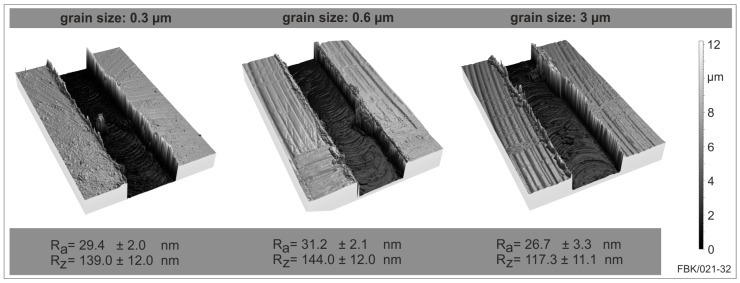
Bottom surface roughness in dependence of the cemented carbide grain size (from left to right: 0.3 µm, 0.6 µm and 3 µm).

The surface roughness shows no significant difference between the three grain sizes micro tools at the beginning of each slot (Figure 14). The arithmetic mean roughness *R*_a_ differs only in the range of 10%, the range in which also the standard deviation varies. The same is true for the average roughness. Despite those small deviations between the grain sizes, it can be stated that both roughness values are lowest for the coarse grain size 3 µm. This can be explained by the wear behavior of the USM-mill. The unworn tool has a minor cutting edge angle of *χ*’_r_ = 12° which reduces the contact of the cutting edge with the slot bottom but on the other side increases the kinematic roughness. When this minor cutting edge angle is reduced by the wear of the minor cutting edge, the kinematic roughness of the slot bottom is reduced, resulting in lower roughness values. As the coarse-grained USM-mill wears rapidly, a lower bottom surface results.

Concluding this case study, the grain size was revealed to be a crucial influencing factor concerning the micro milling process and tool performance. While there was no influence on the resulting bottom surface roughness, a significantly higher tool life and smaller process forces were the result of small grain sizes of the cemented carbide.

## 4. Conclusions

In this paper, state of the art manufacturing of ultra-small micro tools and their application were presented. The investigations covered micro milling with micro end mills and micro grinding with micro pencil grinding tools.

Concerning micro end mills, it was shown that the specifications of the cemented carbide the blank is made of highly influences the quality of the tool as well as its applicability. Sharper, more homogeneous cutting edges without breakouts can be achieved with smaller grain sizes of the cemented carbide. The application of the tools revealed smaller forces and significantly higher tool life with smaller grain sizes.

Considering micro pencil grinding tools, the decisive properties are those of the abrasive layer covering the cemented carbide blank. The case study revealed that larger grit sizes are preferable due to a lower achievable bottom surface roughness of the machined groove as well as higher achievable feed rate and a more stable process behavior. This rather surprising result needs to be further researched. That is, the process parameters as well as the range of grit sizes should be increased and an in depth-analysis of tool wear and tool glazing is necessary to ascertain the material removal mechanisms. The studies on electroless plating of the tools revealed that the increase of grit size is limited. The uniformity of the grit distribution diminishes with rising grit size and at the same time larger grit sizes necessitate longer plating times. The plating time, however, is limited by the lifetime of the plating solution.

For both processes, further studies on cooling and lubrication have to be conducted. It is well known from macro machining that this highly influences the process. Process results in micro machining, such as in [36], show the positive influence of metal working fluids, especially in combination with rough micro tool surfaces where the lubricant’s adhesion tends to be better [37]. 

## Figures and Tables

**Table 1:** Composition of electroless plating solution.

Component	Concentration (g/L)
Nickel sulfate (NiSO_4_·6H_2_O)	30
Sodium hypophosphite (NaH_2_PO_2_)	20
Sodium acetate (C_2_H_3_NaO_2_)	20
Thiourea (CH_4_N_2_S)	0.0004
Hydrochloric acid (HCl)	Adapted to pH-value
Abrasive grits	0–50

**Table 2:** Plating parameters for MPGTs with a 50 µm tool.

Case	Grit Size (µm)	Main Plating Time (Min)	Embedding Time (Min)	Grit Concentration (Solution) (g/L)
(a)	1–2	20 ± 3	0.5–1	0.5–1
(b)	2–4	20 ± 3	1.5–2	4
(c)	3–6	19 ± 3	2–3	8
(d)	5–10	19 ± 3	3–4	15
(e)	6–12	18 ± 3	4	20
(f)	8–16	18 ± 3	5	23

**Table 3:** Parameters used in the case study.

Case	Grit Size (µm)	Feed Rate (mm/min)	Rotational Speed (rpm)	Depth of Cut (µm)	Groove Length (mm)
1	1–2	0.1	30,000	5	1
2	3–6	0.1	30,000	5	1
3	1–2	0.2	50,000	5	1
4	3–6	0.2	50,000	5	1
